# Extracellular Vesicles as Biomarkers for Vascular Disease

**DOI:** 10.3390/biom16040608

**Published:** 2026-04-20

**Authors:** Davide Costa, Michele Andreucci, Nicola Ielapi, Teresa Faga, Antonio Mazza, Giulio Accarino, Umberto Marcello Bracale, Raffaele Serra

**Affiliations:** 1 Interuniversity Center of Phlebolymphology (CIFL), International Research and Educational Program in Clinical and Experimental Biotechnology, University “Magna Graecia” of Catanzaro, 88100 Catanzaro, Italyantonio.mazza5@studenti.unicz.it (A.M.); 2 Department of Medical and Surgical Sciences, University “Magna Graecia” of Catanzaro, 88100 Catanzaro, Italyumbertomarcello.bracale@unina.it (U.M.B.); 3 Department of Public Health, University Federico II of Naples, 80138 Naples, Italy

**Keywords:** vascular disease, extracellular vesicles, aneurysms, venous disease, peripheral artery disease, angiogenesis, chronic wounds, venous thromboembolism, chronic venous disease, proteomics

## Abstract

Vascular diseases (VD) remain a leading global cause of morbidity and mortality, often developing silently before manifesting as severe complications like stroke or ischemia. Traditional diagnostic imaging provides essential anatomical data but frequently fails to capture the dynamic molecular processes underlying vascular pathology. This narrative review summarizes current evidence regarding Extracellular Vesicles (EVs), including exosomes, microvesicles, and apoptotic bodies, as emerging biomarkers and mediators in vascular conditions. The review evaluates the biological mechanisms of EVs across several disorders, including arterial aneurysms, peripheral artery disease, carotid stenosis, and venous thromboembolism. Findings indicate that EVs concentration and molecular cargo, particularly microRNAs and proteins, reflect the physiological state of parent cells, offering a “liquid biopsy” for vascular inflammation, endothelial dysfunction, and plaque vulnerability. Furthermore, the review explores the therapeutic potential of stem cell-derived EVs in promoting angiogenesis and tissue repair in chronic vascular ulcers. Despite these advances, the review concludes that the clinical implementation of EV-based diagnostics faces significant hurdles, primarily due to the lack of standardized isolation and characterization methods. Addressing these methodological challenges is crucial for translating EV research into routine clinical practice.

## 1. Introduction

Vascular disease (VD) represents one of the leading causes of morbidity and mortality worldwide. It includes a wide range of arterial and venous disorders such as arterial aneurysms, peripheral artery disease, carotid stenosis, chronic venous disease, venous thromboembolism, and chronic vascular ulcers. Their incidence is expected to continue increasing due to population aging and the persistence of major risk factors such as diabetes, hypertension, and hyperlipidemia. Many vascular disorders develop silently over years before clinical manifestations appear, often leading to severe complications, including ischemia, thrombosis, or stroke. Consequently, early diagnosis and accurate risk stratification remain major challenges in vascular medicine [1,2].

Current diagnostic approaches rely mainly on imaging techniques and clinical risk assessment. However, imaging primarily provides anatomical information and may not fully capture the dynamic molecular and cellular processes occurring within the vascular wall. For example, in carotid artery disease, the risk of ischemic stroke is increasingly recognized to depend not only on the degree of luminal stenosis but also on plaque composition and vulnerability, which are not always reliably detected by conventional imaging methods [3,4]. More generally, atherosclerosis is a chronic inflammatory disease that can affect multiple vascular beds simultaneously, including the coronary, carotid, peripheral, and aortic arteries, highlighting the need for systemic biomarkers that reflect ongoing vascular pathology [2].

Among emerging candidates, Extracellular Vesicles (EVs) have gained considerable attention as mediators of intercellular communication and as potential biomarkers of vascular disease. EVs are membrane-bound vesicles released by virtually all cell types, including endothelial cells, vascular smooth muscle cells, platelets, and leukocytes. They carry a diverse cargo of proteins, lipids, and nucleic acids, particularly microRNAs, that can be transferred to recipient cells, thereby modulating multiple physiological and pathological processes. Because the molecular composition of EVs reflects the biological state of their parent cells, circulating EVs may provide valuable insights into vascular inflammation, endothelial dysfunction, thrombosis, and vascular remodeling. Recent studies have demonstrated that EV-associated microRNAs may serve as minimally invasive biomarkers for different manifestations of atherosclerotic disease. For instance, specific EV-derived miRNA signatures have been identified in patients with abdominal aortic aneurysm, coronary artery disease, carotid artery stenosis, and peripheral artery disease, suggesting the existence of disease-specific molecular fingerprints detectable in circulation [5,6].

Furthermore, plaque-derived EVs have been shown to contribute to atherosclerotic progression and may help discriminate between stable and vulnerable plaques, highlighting their potential role in risk stratification and disease monitoring [7].

EVs are increasingly studied not only as biomarkers but also as active mediators of vascular disease. This narrative review aims to summarize current evidence on extracellular vesicles as biomarkers in vascular conditions, focusing on arterial aneurysms, peripheral artery disease, carotid stenosis, chronic vascular ulcers, chronic venous disease, and venous thromboembolism. We discuss the biological mechanisms connecting EVs to vascular pathology, their diagnostic and prognostic potential, and the main challenges to their clinical implementation.

## 2. Biology and Classification of Extracellular Vesicles

Extracellular Vesicles (EVs) are heterogeneous membrane-bound structures released by virtually all cell types into the extracellular environment. Initially considered as cellular debris, EVs are now recognized as key mediators of intercellular communication involved in numerous physiological and pathological processes, including immune regulation, angiogenesis, inflammation, and tissue repair. In the vascular system, EVs play an important role in maintaining endothelial homeostasis as well as in mediating vascular injury and remodeling [8,9,10].

EVs are typically classified into three main subtypes according to their size, biogenesis, and release mechanisms: exosomes, microvesicles, and apoptotic bodies. Exosomes are the smallest vesicles, generally ranging from 30 to 150 nm in diameter, and originate from the endosomal pathway. They are formed through the inward budding of multivesicular bodies (MVBs), which subsequently fuse with the plasma membrane to release their vesicular content into the extracellular space. Exosomes contain a variety of bioactive molecules, including proteins, lipids, messenger RNA, and microRNAs, which can be transferred to recipient cells and modulate their biological activity [11,12]. Microvesicles, also referred to as ectosomes, are typically larger vesicles ranging from approximately 100 to 1000 nm in diameter. Unlike exosomes, microvesicles are generated by the outward budding and fission of the plasma membrane. Their formation is often triggered by cellular activation, mechanical stress, or inflammatory stimuli, leading to cytoskeletal rearrangement and membrane remodeling. In vascular diseases, endothelial cells, platelets, leukocytes, and vascular smooth muscle cells are major sources of circulating microvesicles. Apoptotic bodies represent the largest class of EVs, with diameters ranging from 500 nm to several micrometers. These vesicles are released during programmed cell death and contain cellular fragments, including DNA, organelles, and cytoplasmic components [11,12,13]. Although traditionally associated with apoptotic processes, apoptotic bodies may also participate in intercellular signaling and immune modulation [14,15].

It should be noted that, despite differences in biogenesis, exosomes and microvesicles may overlap in size, making their distinction challenging using conventional isolation techniques. As a result, many studies do not clearly separate these subtypes and instead analyze mixed EV populations. This methodological limitation should be considered when interpreting data, particularly in studies attributing specific biological functions or biomarker roles to a given EV subtype [16,17].

The molecular cargo of EVs reflects the physiological or pathological state of their parent cells. EVs can carry a broad spectrum of bioactive molecules, including membrane receptors, signaling proteins, enzymes, lipids, and nucleic acids such as microRNAs, long non-coding RNAs, and fragments of genomic or mitochondrial DNA. This molecular complexity allows EVs to influence multiple biological pathways in recipient cells through the transfer of functional biomolecules [18,19].

In the vascular system, EVs released from endothelial cells, platelets, leukocytes, and smooth muscle cells have been implicated in several key mechanisms underlying vascular pathology [10]. These include endothelial dysfunction, inflammatory signaling, coagulation activation, extracellular matrix degradation, and angiogenic processes. Importantly, EVs can circulate in various biological fluids, including blood, plasma, serum, urine, and saliva, making them attractive candidates for minimally invasive biomarkers [20,21].

Because their concentration, cellular origin, and molecular cargo can change in response to vascular injury or disease, EVs are increasingly investigated as diagnostic and prognostic biomarkers in vascular disorders. Advances in high-throughput technologies such as proteomics, lipidomics, and RNA sequencing have further facilitated the characterization of EV-associated molecular signatures, opening new perspectives for the development of EV-based liquid biopsy approaches in cardiovascular and vascular medicine [5,22,23,24].

A critical aspect of EV research concerns the methodologies used for their isolation, characterization, and quantification. Several techniques are currently employed, each with specific advantages and limitations (Table 1). Ultracentrifugation remains the most widely used method and is considered a reference standard; however, it is time-consuming and may result in co-isolation of non-vesicular components. Size-exclusion chromatography (SEC) provides improved purity and preserves EV integrity but may yield lower concentrations. Precipitation-based methods are simple and scalable but often lack specificity. Immunoaffinity capture allows selective isolation of EV subpopulations based on surface markers, though it is limited by antibody availability and cost. For EV characterization, nanoparticle tracking analysis (NTA), dynamic light scattering (DLS), and flow cytometry are commonly used to determine size distribution and concentration, while Western blotting and omics-based approaches are employed to analyze molecular cargo. Despite these advances, the lack of standardized protocols remains a major limitation in the field, contributing to variability across studies and hindering clinical translation. Future efforts should focus on harmonizing isolation and analytical methods in accordance with international guidelines, such as those proposed by the International Society for Extracellular Vesicles (ISEV) [25,26].

## 3. Extracellular Vesicles in Arterial Aneurysms

Arterial aneurysms, particularly abdominal aortic aneurysm (AAA), represent a major vascular condition characterized by progressive dilation of the arterial wall associated with chronic inflammation, extracellular matrix degradation, and vascular smooth muscle cell dysfunction. Although imaging techniques such as ultrasound and computed tomography remain the standard tools for diagnosis and monitoring, the biological mechanisms driving aneurysm formation and progression are complex and not fully understood. Moreover, aneurysm growth and rupture risk are often difficult to predict using anatomical parameters alone, highlighting the need for molecular biomarkers that reflect the underlying pathophysiological processes [27].

At the pathological level, arterial aneurysms are characterized by progressive weakening and dilation of the vessel wall, primarily driven by chronic inflammation, extracellular matrix degradation, and vascular smooth muscle cell (VSMC) dysfunction. Inflammatory cell infiltration, particularly by macrophages and lymphocytes, leads to the release of cytokines, proteolytic enzymes such as matrix metalloproteinases, and reactive oxygen species. These factors contribute to the breakdown of structural components including elastin and collagen, resulting in reduced mechanical stability of the arterial wall. Concurrently, VSMCs undergo phenotypic switching and apoptosis, further impairing the regenerative capacity of the vessel. Oxidative stress and dysregulated immune responses amplify these processes, promoting aneurysm expansion and increasing the risk of rupture [27].

In recent years, Extracellular Vesicles have emerged as important mediators of intercellular communication within the vascular wall and are increasingly investigated as potential biomarkers in aneurysmal disease. Multiple vascular cell types involved in aneurysm pathogenesis, including endothelial cells, vascular smooth muscle cells, macrophages, and platelets, release EVs in response to inflammatory stimuli, oxidative stress, and mechanical forces. These vesicles can transport bioactive molecules such as proteins, lipids, and nucleic acids that modulate vascular remodeling and inflammatory signaling [28].

Experimental studies suggest that EVs contribute directly to several key mechanisms involved in aneurysm development. For instance, EVs derived from inflammatory cells and vascular smooth muscle cells can promote extracellular matrix degradation by transferring matrix metalloproteinases (MMPs) and other proteolytic enzymes. This process contributes to weakening of the aortic wall, a hallmark of aneurysm formation. In addition, EVs may influence vascular smooth muscle cell apoptosis and phenotypic switching, further destabilizing the structural integrity of the vessel wall [20,28,29,30,31].

Circulating EVs have also attracted interest as minimally invasive biomarkers for aneurysmal disease. Alterations in the concentration and cellular origin of EVs have been reported in patients with abdominal aortic aneurysm compared with healthy individuals. In particular, increased levels of endothelial- and platelet-derived EVs have been associated with vascular inflammation and endothelial dysfunction, suggesting that EV profiles may reflect disease activity [28,32].

Another promising area of investigation concerns the molecular cargo of EVs, particularly microRNAs (miRNAs). EV-associated miRNAs have been implicated in the regulation of pathways related to inflammation, extracellular matrix remodeling, and vascular smooth muscle cell behavior. Several studies have identified specific EV-derived miRNA signatures associated with aneurysm presence and progression, raising the possibility that circulating EV-miRNAs could serve as diagnostic or prognostic biomarkers. Furthermore, EV-based molecular profiling may provide insights into the biological mechanisms driving arterial aneurysm, including cerebral aneurysms, expansion, and rupture risk [33,34,35,36].

In the context of aortic aneurysms, EVs released from immune cells, endothelial cells, vascular smooth muscle cells (VSMCs), and other circulating sources influence cellular interactions within the aortic wall. These vesicles can transfer bioactive molecules that regulate signaling pathways involved in inflammation, apoptosis, oxidative stress, and matrix degradation. One of the key mechanisms through which EVs contribute to aneurysm pathology involves their ability to modulate immune cell behavior. For example, EVs derived from activated T lymphocytes have been shown to aggravate abdominal aortic aneurysm (AAA) development by influencing macrophage function. These T cell–derived EVs transport specific metabolic enzymes and signaling molecules, including pyruvate kinase muscle isozyme 2 (PKM2), which can reprogram macrophage metabolism and enhance lipid peroxidation. The transfer of PKM2 promotes oxidative stress and increases the migratory capacity of macrophages, thereby amplifying inflammatory responses within the aneurysmal aortic wall. Macrophage infiltration is a central event in aneurysm progression, as these cells release inflammatory cytokines, reactive oxygen species, and matrix metalloproteinases that degrade structural components such as elastin and collagen. Through EV-mediated communication, T lymphocytes can therefore indirectly intensify matrix destruction and vascular wall weakening, facilitating aneurysm expansion. Lipid peroxidation induced by these vesicles also contributes to ferroptosis-like processes and further inflammatory activation, creating a self-perpetuating cycle of oxidative damage and immune cell recruitment. In addition to immune cell–derived vesicles, circulating exosomes originating from multiple tissues may reflect pathological changes associated with aneurysm development [37,38]. Proteomic analyses of serum exosomes in experimental models of thoracic aortic aneurysm (TAA) reveal substantial alterations in the abundance of proteins involved in inflammation, metabolic regulation, and extracellular matrix organization. Label-free quantitative proteomic studies have identified numerous differentially expressed proteins in exosomes isolated from mice with TAA compared with healthy controls. Many of these proteins are associated with pathways relevant to aneurysm pathogenesis, including oxidative stress responses, cytoskeletal organization, complement activation, and lipid metabolism. For instance, proteins related to inflammatory signaling and immune activation are frequently enriched in aneurysm-associated exosomes, suggesting that systemic EV populations reflect ongoing vascular inflammation. Other proteins linked to extracellular matrix turnover and cell adhesion indicate that exosomes may participate in remodeling processes occurring within the aortic wall. These findings support the concept that circulating exosomes may serve as minimally invasive biomarkers for early detection and monitoring of aneurysm progression. Because EVs protect their molecular cargo from degradation in the bloodstream, they provide a stable reservoir of disease-related molecules that can be detected through proteomic or transcriptomic profiling. Beyond aneurysms specifically, broader research on extracellular vesicles in cardiovascular disease underscores their multifaceted roles in vascular pathology. EVs participate in endothelial dysfunction, atherosclerosis, myocardial injury, and vascular inflammation through mechanisms that overlap with those driving aneurysm formation. They can regulate endothelial permeability, modulate smooth muscle cell proliferation and apoptosis, and influence the balance between pro-inflammatory and anti-inflammatory immune responses. For example, EVs derived from endothelial cells under conditions of oxidative stress or mechanical injury can carry microRNAs and proteins that promote inflammatory signaling in neighboring cells. Similarly, VSMC-derived vesicles may alter extracellular matrix composition by transporting enzymes that degrade structural proteins or by influencing fibroblast activity. In aneurysmal disease, the degeneration of elastic fibers and the loss of VSMC integrity are key pathological features, and EV-mediated signaling likely contributes to both processes. EV-derived microRNAs regulate matrix remodeling, apoptosis, and inflammation, contributing to smooth muscle cell phenotypic switching and aortic wall weakening. Another important aspect of EV biology in cardiovascular disease is their potential role in oxidative stress propagation. Vesicles can carry enzymes or signaling molecules that enhance the production of reactive oxygen species in recipient cells, thereby exacerbating tissue damage and inflammation. This is particularly relevant in aneurysms, where oxidative stress contributes to endothelial dysfunction, smooth muscle cell apoptosis, and activation of matrix-degrading proteases. In addition to their pathogenic roles, EVs are increasingly being explored as therapeutic tools because of their natural ability to deliver bioactive molecules to specific cell types. Engineered or stem cell–derived EVs have shown promise in experimental models of cardiovascular disease by reducing inflammation, promoting tissue repair, and modulating immune responses. Their small size, biological compatibility, and capacity to cross biological barriers make them attractive candidates for cell-free therapies. For aneurysm treatment, EV-based strategies could potentially be designed to deliver anti-inflammatory molecules, microRNAs that inhibit matrix degradation, or proteins that support vascular wall stability. However, significant challenges remain before clinical application can be realized, including standardization of EV isolation methods, precise characterization of vesicle subtypes, and improved understanding of their biodistribution and safety profiles. Overall, accumulating evidence indicates that extracellular vesicles are central players in the pathophysiology of aortic aneurysms. They act as carriers of molecular signals that link immune activation, oxidative stress, metabolic reprogramming, and extracellular matrix degradation within the vascular wall. T lymphocyte–derived vesicles that enhance macrophage lipid peroxidation and migration illustrate how immune cell communication via EVs can accelerate aneurysm progression. Meanwhile, proteomic profiling of circulating exosomes provides insight into systemic molecular changes associated with thoracic aneurysm formation and highlights their potential value as diagnostic biomarkers. Taken together, these studies emphasize that EVs are not merely byproducts of cellular activity but active regulators of disease processes. Understanding the complex signaling networks mediated by extracellular vesicles may therefore open new avenues for early diagnosis, risk stratification, and targeted therapy in patients with aortic aneurysms and other cardiovascular disorders [38,39,40,41].

Despite these promising findings, several challenges remain before EVs can be translated into clinical biomarkers for aneurysmal disease. These include the lack of standardized methods for EV isolation and characterization, variability between analytical platforms, and limited validation in large prospective cohorts. Nevertheless, the growing body of evidence suggests that EVs represent a promising tool for improving our understanding of aneurysm biology and for developing novel biomarker strategies in vascular medicine [41,42].

In contrast, some studies highlight the important but contrasting roles of EVs in the development and progression of abdominal aortic aneurysm (AAA), emphasizing how different immune cell–derived vesicles can either exacerbate or mitigate vascular inflammation and tissue remodeling. For example, Ma et al. [43] investigates the protective role of EVs derived from M2 macrophages in AAA. Using an angiotensin II–induced aneurysm model in ApoE−/− mice, the authors demonstrate that EVs released by M2 macrophages contain regulatory microRNAs, particularly miR-221-5p, which play a key role in modulating macrophage polarization and inflammatory responses in the aortic wall. Administration of these EVs significantly reduced aortic dilation, inflammatory cell infiltration, and extracellular matrix degradation in experimental models. Mechanistically, miR-221-5p was shown to regulate the PARP-1/PP-1α/JNK/c-Jun signaling pathway, which is involved in inflammatory activation and macrophage phenotype regulation. Through this pathway, M2 macrophage-derived EVs promote an anti-inflammatory macrophage phenotype and suppress processes that contribute to vascular wall degeneration and aneurysm progression. The study therefore suggests that EV-mediated transfer of regulatory microRNAs represents an important mechanism of intercellular communication within the vascular microenvironment. These findings also indicate that EVs derived from anti-inflammatory macrophages may represent a potential therapeutic strategy for limiting AAA progression. Supporting this concept, altered activity of the PARP-1 pathway was also observed in human aneurysmal tissue samples, suggesting that the molecular mechanisms identified in experimental models may be relevant to human disease.

Chen et al. [44] also emphasizes a protective mechanism mediated by EVs in aneurysm development. The authors focus on EV-mediated regulation of macrophage oxidative stress and migration in the context of AAA. Through experimental models and cellular analyses, the study demonstrates that EV-related molecular pathways influence macrophage lipid metabolism, oxidative balance, and inflammatory behavior within the aortic wall. By modulating processes such as lipid peroxidation and macrophage recruitment, EV-associated signaling contributes to maintaining vascular homeostasis and limiting excessive inflammatory damage. The study highlights how alterations in EV content and signaling pathways can significantly influence the cellular environment of the aneurysmal aorta. In particular, EV-mediated regulation of macrophage activity appears to be a key factor controlling the balance between inflammatory tissue damage and protective immune responses during aneurysm development.

Together, these findings demonstrate that extracellular vesicles act as critical mediators of intercellular communication in the aneurysmal microenvironment. By transferring microRNAs, enzymes, and other signaling molecules between immune and vascular cells, EVs regulate macrophage polarization, oxidative stress, and inflammatory activity, all of which play central roles in aneurysm progression. The protective effects observed in these studies suggest that EVs may serve not only as biomarkers of disease progression but also as promising therapeutic tools. Targeting EV-mediated signaling pathways or harnessing beneficial EV populations could therefore represent an innovative strategy for modulating inflammation and preventing the development or progression of abdominal aortic aneurysms [43,44].

## 4. Extracellular Vesicles in Peripheral Artery Disease

Peripheral artery disease (PAD) is a common manifestation of systemic atherosclerosis characterized by progressive narrowing and occlusion of arteries supplying the lower extremities. The disease affects millions of individuals worldwide and is associated with significant morbidity, including claudication, critical limb ischemia, impaired wound healing, and increased cardiovascular mortality. The pathophysiology of PAD involves complex interactions among endothelial dysfunction, chronic inflammation, lipid accumulation, thrombosis, and impaired angiogenesis. Despite advances in diagnostic imaging and clinical management, early detection and accurate monitoring of disease progression remain challenging [45].

At the pathological level, PAD is driven by progressive atherosclerotic plaque formation characterized by lipid accumulation, endothelial dysfunction, and chronic inflammation. Endothelial injury promotes leukocyte adhesion and infiltration, leading to cytokine release and oxidative stress. This inflammatory environment contributes to smooth muscle cell proliferation and extracellular matrix remodeling, ultimately resulting in arterial narrowing and impaired perfusion. In advanced stages, reduced oxygen supply leads to ischemia, tissue damage, and defective angiogenic responses [45].

EVs have emerged as potential biomarkers reflecting the cellular and molecular processes underlying PAD. Circulating EVs are released by multiple vascular cell types involved in atherosclerotic disease, including endothelial cells, platelets, leukocytes, and vascular smooth muscle cells. These vesicles carry bioactive cargo such as proteins, lipids, and nucleic acids that can influence vascular inflammation, coagulation pathways, and angiogenic responses [46,47].

Several studies have reported increased levels of circulating EVs in patients with PAD compared with healthy individuals. In particular, elevated concentrations of endothelial-derived and platelet-derived EVs have been associated with endothelial injury, platelet activation, and prothrombotic states commonly observed in PAD. These EV populations may therefore serve as indicators of vascular dysfunction and systemic inflammatory activity. Moreover, the levels of specific EV subtypes have been correlated with disease severity, suggesting a potential role for EV profiling in risk stratification [5].

Beyond their diagnostic potential, EVs may also contribute to the pathophysiology of PAD. Experimental evidence indicates that EVs can modulate angiogenesis, a process that is critically impaired in advanced stages of PAD. For example, EVs derived from endothelial progenitor cells (EPCs) or mesenchymal stem cells have been shown to promote angiogenic signaling and tissue repair in ischemic tissues. Conversely, EVs released under inflammatory or oxidative stress conditions may carry molecules that inhibit endothelial regeneration or promote vascular dysfunction [48,49]. In particular, Saenz-Pipaon et al. [46] performed transcriptomic analysis that revealed changes in gene expression patterns within EVs derived from PAD patients. Among the molecules identified, the inflammatory protein calprotectin emerged as a significant candidate biomarker. In fact, calprotectin is a known mediator of vascular inflammation and immune activation. Elevated levels of this protein have previously been linked to inflammatory diseases and cardiovascular pathology. The study demonstrated that EVs from PAD patients contained higher levels of calprotectin, and importantly, these levels were associated with clinical outcomes and disease prognosis.

Particular attention has been devoted to the role of EV-associated microRNAs in PAD. MicroRNAs transported by EVs can regulate gene expression in recipient cells and influence pathways related to endothelial function, vascular remodeling, and inflammation. Alterations in specific EV-derived microRNA profiles have been reported in patients with PAD and may reflect ongoing vascular injury or ischemic adaptation. These findings support the concept that EV-associated molecular signatures could serve as minimally invasive biomarkers for disease detection and progression monitoring [50,51].

The study by Wei et al. [52] investigates whether EV-associated R-Ras could serve as a biomarker for PAD. R-Ras is a small GTPase involved in vascular stability, endothelial function, and angiogenesis, processes that are critically impaired in PAD. The authors analyzed circulating EVs isolated from patients with PAD and healthy controls. They found that R-Ras protein levels in EVs were significantly altered in PAD patients, suggesting a disease-associated shift in vesicle cargo. Importantly, the EV-associated R-Ras levels correlated with clinical indicators of PAD severity, indicating that this molecule may reflect underlying vascular dysfunction. Mechanistically, R-Ras plays a role in maintaining vascular integrity and regulating endothelial cell behavior. Reduced or dysregulated R-Ras signaling has been associated with impaired angiogenesis and abnormal vascular remodeling, two features characteristic of PAD. The presence of R-Ras in circulating EVs therefore likely reflects vascular cell stress or altered endothelial signaling during disease progression. These results suggest that EV-associated R-Ras could serve as a minimally invasive biomarker for PAD diagnosis or monitoring, though the authors emphasize that further validation in larger patient cohorts is required.

Despite these promising observations, several challenges remain in translating EV research into clinical practice. Standardization of EV isolation techniques, identification of reliable cell-specific markers, and validation of EV-based biomarkers in large clinical cohorts are necessary steps for their implementation in vascular medicine. Nevertheless, the growing body of evidence suggests that EVs may provide valuable insights into the biological mechanisms of PAD and hold significant potential for improving diagnostic and prognostic strategies in patients with peripheral arterial disease [53].

## 5. Extracellular Vesicles in Carotid Stenosis

Carotid artery stenosis is a major cause of ischemic stroke and represents an important manifestation of systemic atherosclerotic disease. The condition is characterized by progressive narrowing of the carotid arteries due to the formation of atherosclerotic plaques within the arterial wall. While the degree of luminal stenosis has traditionally been used to guide clinical decision-making, it is now widely recognized that the risk of cerebrovascular events is strongly influenced by plaque composition and vulnerability rather than stenosis severity alone. Features such as inflammation, lipid-rich necrotic cores, intraplaque hemorrhage, and fibrous cap thinning contribute to plaque instability and increase the likelihood of embolization and stroke [54].

Carotid artery stenosis arises from the development of atherosclerotic plaques within the carotid arterial wall, driven by endothelial dysfunction, lipid accumulation, and chronic inflammation. The pathological process begins with endothelial injury, which facilitates the infiltration of low-density lipoproteins and recruitment of inflammatory cells, particularly monocytes that differentiate into macrophages. These cells internalize lipids and form foam cells, contributing to plaque growth. Over time, the plaque evolves into a complex structure with a lipid-rich necrotic core, fibrous cap, and inflammatory infiltrate. Plaque instability, rather than luminal narrowing alone, is a critical determinant of clinical risk and is associated with thinning of the fibrous cap, increased proteolytic activity, neovascularization, and intraplaque hemorrhage. These features increase the likelihood of plaque rupture and embolization, leading to ischemic cerebrovascular events [54].

EVs have emerged as potential mediators and biomarkers of carotid plaque development and instability. Atherosclerotic plaques contain multiple cell types, including endothelial cells, macrophages, smooth muscle cells, and platelets, that actively release EVs during inflammatory activation and cellular stress. These vesicles can transfer bioactive molecules to neighboring cells, thereby contributing to intercellular communication within the plaque microenvironment. Experimental studies have demonstrated that EVs derived from atherosclerotic plaques may actively participate in plaque progression. For example, EVs released by macrophages and smooth muscle cells can promote inflammatory signaling, extracellular matrix degradation, and vascular remodeling. Some EV populations have also been shown to carry matrix metalloproteinases and pro-inflammatory mediators capable of weakening the fibrous cap and increasing plaque vulnerability. In addition, EVs may contribute to endothelial dysfunction and thrombogenic processes that further enhance the risk of cerebrovascular events [48,49,55,56].

Circulating EVs have therefore attracted increasing interest as minimally invasive biomarkers for carotid atherosclerosis. Several studies have reported elevated levels of endothelial-derived and platelet-derived EVs in patients with carotid artery disease. These vesicles are thought to reflect endothelial injury, platelet activation, and ongoing inflammatory activity within the vascular wall. Moreover, specific EV subtypes have been associated with symptomatic carotid stenosis and recent cerebrovascular events, suggesting a potential role in identifying high-risk patients [57].

Another promising area of investigation involves the molecular cargo of EVs, particularly microRNAs and other regulatory nucleic acids. EV-associated microRNAs have been implicated in the regulation of pathways related to inflammation, lipid metabolism, and vascular remodeling. Alterations in EV-derived microRNA profiles have been observed in patients with carotid atherosclerosis and may help distinguish between stable and unstable plaques. This raises the possibility that EV-based molecular signatures could complement imaging techniques in the assessment of plaque vulnerability and stroke risk [7].

Despite the growing interest in EVs as biomarkers in carotid artery disease, several limitations remain. Variability in EV isolation methods, lack of standardized quantification techniques, and heterogeneity among patient populations complicate comparisons between studies. Larger prospective investigations are needed to determine whether EV profiling can improve clinical risk stratification and guide therapeutic decision-making in patients with carotid stenosis [57,58].

## 6. Extracellular Vesicles in Chronic Venous Disease

Chronic venous disease (CVD) is a highly prevalent vascular disorder encompassing a spectrum of conditions ranging from telangiectasia and varicose veins to chronic venous insufficiency and venous leg ulcers. The disease is characterized by venous hypertension, valvular dysfunction, and progressive alterations of the venous wall and microcirculation. Persistent venous hypertension leads to endothelial activation, leukocyte recruitment, inflammation, and structural remodeling of the venous wall. These processes contribute to symptoms such as edema, skin changes, and in advanced stages, the development of venous ulceration [59].

The pathophysiology of chronic venous disease is primarily driven by sustained venous hypertension resulting from valvular incompetence and impaired venous return. Elevated venous pressure leads to endothelial activation and dysfunction, which promotes leukocyte adhesion, migration, and the release of pro-inflammatory mediators. This inflammatory cascade contributes to structural alterations in the venous wall, including extracellular matrix remodeling, smooth muscle cell dysfunction, and progressive vein dilation. Microcirculatory changes, such as capillary leakage and reduced oxygen diffusion, further exacerbate tissue hypoxia and inflammation. Over time, these processes lead to skin changes, fibrosis, and in advanced stages, the development of venous ulcers. The chronic inflammatory state and impaired tissue repair mechanisms are central features of disease progression [60].

EVs have emerged as potential mediators and biomarkers of venous pathology. Cells involved in the pathophysiology of chronic venous disease, including endothelial cells, leukocytes, platelets, and smooth muscle cells, are capable of releasing EVs in response to inflammatory stimuli, mechanical stress, and hypoxic conditions. These vesicles carry bioactive cargo such as proteins, lipids, cytokines, and nucleic acids that can influence cellular communication within the vascular wall and surrounding tissues [60,61]. Moreover, increased concentrations of endothelial-derived EVs have been associated with endothelial dysfunction and venous hypertension. Platelet-derived EVs have also been observed in higher levels in individuals with advanced disease, reflecting platelet activation and a pro-inflammatory microenvironment. These changes suggest that EV profiles may reflect disease severity and ongoing vascular injury in CVD [62,63].

The study by Ortega et al. [64] indicates that the molecular machinery associated with EV production and cellular stress is upregulated in placentas from pregnancies complicated by CVD. Since extracellular vesicles mediate communication between maternal and fetal tissues, changes in their production or composition may influence inflammatory signaling, vascular remodeling, and tissue adaptation during pregnancy. Overall, the study provides evidence that chronic venous disease during pregnancy is associated with molecular alterations in placental tissue, particularly in proteins related to extracellular vesicles and stress regulation. These findings support the idea that maternal venous pathology may contribute to placental dysfunction and highlight extracellular vesicle–related proteins as potential indicators of placental stress in CVD pregnancies.

Despite the growing interest in EVs in CVD, the available evidence remains relatively limited compared with arterial vascular disorders. Nevertheless, EV research offers promising insights into the molecular mechanisms underlying venous pathology and may contribute to the development of novel diagnostic and prognostic strategies for chronic venous disease.

## 7. Extracellular Vesicles in Venous Thromboembolism

Venous thromboembolism (VTE), which includes deep vein thrombosis and pulmonary embolism, represents a major cause of morbidity and mortality worldwide. The pathogenesis of VTE is traditionally explained by Virchow’s triad, which includes venous stasis, endothelial injury, and hypercoagulability. These factors lead to the activation of coagulation pathways, platelet aggregation, and thrombus formation within the venous circulation. Although several clinical risk factors for VTE have been identified, predicting thrombotic events and assessing recurrence risk remain significant challenges [65].

Venous thromboembolism develops as a result of the interplay between venous stasis, endothelial injury, and hypercoagulability, collectively known as Virchow’s triad. Reduced blood flow, often due to immobility or venous obstruction, promotes the accumulation of coagulation factors and facilitates thrombus formation. Endothelial dysfunction further contributes by exposing procoagulant surfaces and reducing the production of anticoagulant and fibrinolytic factors. In parallel, systemic or localized hypercoagulable states, arising from genetic predisposition, malignancy, inflammation, or hormonal influences, enhance thrombin generation and fibrin deposition. The resulting thrombus is composed of fibrin, platelets, and trapped blood cells, which can propagate within the venous system and potentially embolize to the pulmonary circulation. Inflammatory signaling and interactions between coagulation and immune pathways play a critical role in both thrombus formation and resolution [66].

Extracellular Vesicles have gained increasing attention as contributors to thrombotic processes and as potential biomarkers of venous thrombosis. EVs derived from platelets, endothelial cells, leukocytes, and red blood cells can exhibit procoagulant properties by exposing negatively charged phospholipids and tissue factor on their surface. These characteristics allow EVs to provide a catalytic surface that enhances the activation of coagulation factors and promotes thrombin generation. Platelet-derived EVs represent one of the most abundant EV populations in circulation and are strongly associated with coagulation activation. During platelet activation, large quantities of EVs are released, carrying procoagulant molecules that contribute to thrombus propagation. Similarly, endothelial-derived EVs released during vascular injury or inflammation may amplify coagulation signaling and promote thrombogenesis [62,63].

In patients with venous thromboembolism, elevated levels of circulating EVs with procoagulant activity have been reported [67]. These vesicles may carry tissue factor and other prothrombotic molecules capable of accelerating clot formation. Consequently, EV-associated tissue factor activity has been proposed as a potential biomarker for thrombotic risk assessment [68]. In addition, EV-derived microRNAs involved in coagulation and inflammatory pathways have been investigated as potential indicators of thrombotic disease [69].

Beyond their role in thrombus formation, EVs may also participate in thrombus resolution and vascular repair. Some EV populations can modulate fibrinolysis, endothelial regeneration, and inflammatory responses within the thrombotic environment. These findings highlight the complex and multifaceted role of EVs in the pathophysiology of venous thromboembolism [70].

The study by Charles et al. [71], conducted in patients with newly diagnosed multiple myeloma, evaluated circulating TF-bearing EVs together with procoagulant phospholipids (PPL) and D-dimer as potential predictors of VTE. Blood samples were collected at diagnosis and analyzed using functional coagulation assays. The results showed that patients who later developed VTE had significantly higher levels of TF-positive EV activity and procoagulant phospholipids compared with those who did not experience thrombotic events. Elevated D-dimer levels were also associated with increased thrombotic risk. The authors concluded that a combination of these biomarkers may improve risk stratification for VTE in multiple myeloma patients.

The study by van Es et al. [72] used a prospective cohort design to determine whether TF-exposing EVs could predict VTE in patients with various cancers. Plasma samples were analyzed for EV-associated TF activity and patients were followed for the occurrence of thrombotic events. Although cancer patients showed detectable TF-positive EVs, the study found limited predictive value for future VTE. While higher TF-EV activity was associated with cancer-related coagulation activation, it did not reliably identify individuals who would develop thrombosis.

Despite these promising insights, the clinical application of EVs as biomarkers in VTE remains limited by methodological challenges, including heterogeneity in EV detection methods and variability in patient populations. Future large-scale studies are needed to clarify the diagnostic and prognostic value of EVs in venous thrombosis and to determine whether EV-based biomarkers could improve current strategies for VTE risk assessment and management.

## 8. Extracellular Vesicles in Chronic Vascular Ulcers

Chronic vascular ulcers represent a severe complication of both arterial and venous diseases and are characterized by impaired wound healing, persistent inflammation, and microvascular dysfunction. These lesions commonly occur in patients with peripheral artery disease, chronic venous insufficiency, diabetes, or mixed vascular etiologies. The pathophysiology of chronic ulcers involves complex interactions between ischemia, inflammation, extracellular matrix remodeling, and impaired angiogenesis, all of which contribute to delayed tissue repair [73].

Chronic vascular ulcers result from a failure of normal wound healing processes in the context of impaired perfusion, persistent inflammation, and microvascular dysfunction. In arterial ulcers, reduced blood supply leads to tissue ischemia, hypoxia, and necrosis, limiting the delivery of oxygen and nutrients required for repair. In venous ulcers, sustained venous hypertension causes capillary damage, increased permeability, and inflammatory cell infiltration, leading to tissue edema and fibrosis. Regardless of etiology, chronic ulcers are characterized by prolonged inflammatory activity, impaired angiogenesis, and defective extracellular matrix remodeling. Dysregulation of growth factors, cytokines, and proteolytic enzymes further disrupts the balance between tissue degradation and regeneration. This pathological environment prevents progression through the normal stages of wound healing, resulting in persistent, non-healing lesions [73].

In recent years, Extracellular Vesicles have attracted increasing attention as mediators of tissue repair and potential therapeutic tools in chronic wound healing. EVs released by various cell types, including endothelial cells, fibroblasts, immune cells, and stem cells, play an important role in regulating cellular communication within the wound microenvironment. These vesicles carry bioactive molecules such as growth factors, cytokines, proteins, and nucleic acids that can modulate multiple stages of the healing process [74,75].

One of the most relevant mechanisms through which EVs contribute to wound healing is the regulation of angiogenesis. EVs derived from EPCs and mesenchymal stem cells have been shown to promote endothelial cell proliferation, migration, and the formation of new microvascular networks. This angiogenic activity is particularly important in ischemic tissues, where the restoration of adequate blood supply is essential for effective wound repair [74,76,77,78,79,80].

EV-associated microRNAs also play a significant role in modulating inflammatory responses and tissue regeneration in chronic wounds. Several EV-derived microRNAs have been implicated in the regulation of pathways involved in angiogenesis, collagen synthesis, and fibroblast activation. By transferring these regulatory molecules to recipient cells, EVs can influence the balance between inflammation and tissue repair within the wound environment [81,82].

In addition to their regenerative properties, EVs may also serve as biomarkers for wound progression and healing outcomes. Alterations in circulating EV profiles have been observed in patients with chronic ulcers, reflecting ongoing inflammation, endothelial dysfunction, and impaired microcirculation. These findings suggest that EV-based biomarkers could potentially assist clinicians in monitoring wound status, wound care, and predicting healing responses [83].

In the context of diabetic foot ulcers (DFUs), adipose tissue is no longer viewed merely as fat storage but as an endocrine organ rich in stem cells, the so-called Adipose-Derived Stem Cells (ADSCs). ADSC-derived EVs are particularly promising for DFU treatment due to their accessibility and potent regenerative properties. In fact, ADSC-EVs promote healing by enhancing the proliferation and migration of keratinocytes and fibroblasts, which are essential for re-epithelialization. Also, they carry pro-angiogenic factors that stimulate vascular endothelial cells to form new blood vessels, counteracting the ischemia characteristic of diabetic wounds. Moreover, the therapeutic potential of these EVs is largely attributed to their miRNA cargo, which can uniquely regulate gene expression in target cells to restore metabolic homeostasis. Recent research has identified specific molecular pathways through which EVs influence diabetic healing. A critical discovery involves miR-ERIA, a novel miRNA enriched in macrophage-derived EVs under hyperglycemic conditions (specifically after exposure to Advanced Glycation End products, or AGEs) [84].

While much research is preclinical, a pioneering pilot case–control study has demonstrated the clinical effectiveness of autologous serum-derived EVs (s-EVs) for Chronic venous ulcers (CVUs) that failed to respond to conventional treatments. The efficacy of s-EVs is linked to their enrichment in Transforming Growth Factor-beta 1 (TGFβ1), which induces microvascular proliferation and tissue repair [85].

Despite these promising developments, several challenges remain before EV-based therapies can be translated into clinical practice. Standardization of EV isolation techniques, optimization of delivery methods, and large-scale clinical studies are required to validate the safety and efficacy of EV-based therapeutic strategies. Nevertheless, EV research represents a promising frontier in the development of novel regenerative approaches for chronic vascular ulcers [85,86].

## 9. Future Perspectives and Clinical Translation

A key aspect in evaluating EVs as biomarkers lies in identifying specific molecular cargo associated with disease processes. EVs carry a wide range of bioactive molecules, including proteins (e.g., tissue factor, matrix metalloproteinases), lipids, and nucleic acids such as microRNAs. Among these, EV-associated microRNAs have received particular attention due to their stability in circulation and their role in gene regulation [87].

A comprehensive overview of the main EV-associated cargos and their roles in different vascular diseases is summarized in Table 2.

Different vascular diseases appear to be associated with distinct EV cargo profiles. For example, miRNAs involved in inflammation and extracellular matrix remodeling are frequently reported in aneurysmal disease, whereas procoagulant proteins such as tissue factor are more relevant in venous thromboembolism. Similarly, proteins related to endothelial dysfunction and angiogenesis have been identified in PAD and carotid atherosclerosis [88].

However, the clinical utility of these molecules varies considerably. While some EV-associated biomarkers demonstrate strong associations with disease presence or severity, their specificity may be limited due to overlap between different vascular conditions. In addition, technical variability and lack of standardized quantification methods complicate their validation. A systematic comparison of EV cargo across vascular diseases, including their diagnostic performance, strengths, and limitations, is therefore essential for advancing their clinical application [89].

To better contextualize the clinical relevance of extracellular vesicles as biomarkers, it is important to consider key parameters of biomarker performance, including sensitivity, specificity, reproducibility, and predictive value. Although numerous studies have demonstrated associations between EV levels or cargo and vascular diseases, only a limited number have systematically evaluated their diagnostic accuracy or compared them with established clinical biomarkers. Furthermore, variability in EV isolation techniques and analytical platforms significantly affects measurement reproducibility, thereby limiting cross-study comparability [90].

Consequently, while EVs hold promise as minimally invasive “liquid biopsy” tools, their clinical applicability depends on rigorous validation in large-scale studies, including receiver operating characteristic (ROC) curve analyses and standardization of pre-analytical and analytical procedures. Addressing these aspects is essential to determine whether EV-based biomarkers can provide incremental value over current diagnostic strategies in vascular medicine [91].

A key limitation of EVs as biomarkers is their uncertain diagnostic performance and clinical utility. While a substantial body of literature has demonstrated associations between EV concentration, cellular origin, or molecular cargo and various vascular pathologies, significantly less attention has been devoted to systematically evaluating key biomarker characteristics such as sensitivity, specificity, predictive value, and reproducibility. These parameters are essential for determining whether EV-based assays can meaningfully improve current diagnostic and prognostic strategies. In many studies, EV alterations are reported in relation to disease presence or severity; however, these findings are often derived from relatively small cohorts, lack external validation, and are not consistently compared against established clinical or imaging-based biomarkers. As a result, it remains unclear whether EVs provide incremental diagnostic value or merely reflect known pathological processes already captured by conventional methods [92].

Another important limitation relates to the heterogeneity of EV populations and the variability introduced by pre-analytical and analytical procedures. Factors such as sample type (plasma versus serum), collection protocols, storage conditions, and isolation techniques can significantly influence EV yield and composition. This variability directly impacts the reproducibility of results and complicates cross-study comparisons. Moreover, different analytical platforms, including NTA, flow cytometry, and omics-based approaches, may yield divergent quantitative and qualitative data, further challenging the standardization required for clinical application [93].

From a clinical perspective, the specificity of EV-derived biomarkers also represents a significant challenge. Many EV-associated molecules, particularly microRNAs and inflammatory proteins, are not unique to a single vascular condition but are shared across multiple cardiovascular and systemic diseases. This overlap reduces their discriminatory power and may lead to false-positive findings when applied in heterogeneous patient populations. Therefore, rather than relying on single EV-derived markers, future research should focus on the development of multi-marker panels or integrated molecular signatures that combine several EV components to improve diagnostic accuracy. Such approaches may enhance sensitivity and specificity by capturing the complex and multifactorial nature of vascular diseases [94].

Despite the promising experimental findings, it is important to emphasize that most evidence regarding EV-based diagnostics and therapies derives from in vitro studies or preclinical models. Translation into clinical practice remains limited, and significant challenges must be overcome before EVs can be integrated into routine vascular medicine. These include standardization of isolation techniques, validation in large and diverse patient cohorts, and demonstration of clear superiority or added value compared to existing diagnostic tools [95].

Moreover, the biological complexity and heterogeneity of EV populations pose additional challenges for their clinical use. As a result, EV-based precision diagnostics and therapeutic strategies, while highly promising, should currently be considered an emerging field that requires further rigorous investigation before widespread clinical implementation [96].

The growing body of evidence surrounding EVs highlights their potential as both biomarkers and therapeutic mediators in vascular diseases. Advances in molecular biology, high-throughput sequencing technologies, and nanomedicine have significantly improved our understanding of EV biogenesis, molecular cargo, and functional roles in intercellular communication. These developments have opened new perspectives for the use of EVs as minimally invasive diagnostic tools in vascular medicine [95,97,98].

One of the most promising applications of EVs lies in their use as circulating biomarkers for disease detection and risk stratification. Because EVs carry molecular signatures reflecting the physiological state of their parent cells, they may provide real-time information about ongoing pathological processes within the vascular system. EV-derived proteins, lipids, and nucleic acids, including microRNAs, have been investigated as potential biomarkers for several vascular conditions, including atherosclerosis, aneurysmal disease, peripheral artery disease, and venous thrombosis [93,99,100,101].

Another emerging field involves the concept of EV-based “liquid biopsy.” By analyzing the molecular cargo of circulating EVs, clinicians may be able to detect early vascular dysfunction before structural changes become evident through conventional imaging techniques. This approach could significantly improve early diagnosis and enable more personalized management strategies for patients with vascular diseases [23,102,103].

Beyond their diagnostic potential, EVs also hold promise as therapeutic agents or drug delivery systems. Due to their natural ability to transfer biological molecules between cells, EVs can be engineered to deliver specific therapeutic cargo, such as regulatory RNAs or anti-inflammatory molecules, directly to target tissues. In experimental models, EVs derived from stem cells have demonstrated regenerative and angiogenic properties that may be beneficial in ischemic vascular diseases and chronic wounds [84,85,104].

However, several technical and methodological challenges must be addressed before EV-based approaches can be widely implemented in clinical practice. These include the lack of standardized protocols for EV isolation and characterization, variability in analytical techniques, and difficulties in identifying cell-specific EV populations. Furthermore, large prospective clinical studies are needed to validate EV-based biomarkers and determine their clinical utility [105,106].

**Figure 1:**
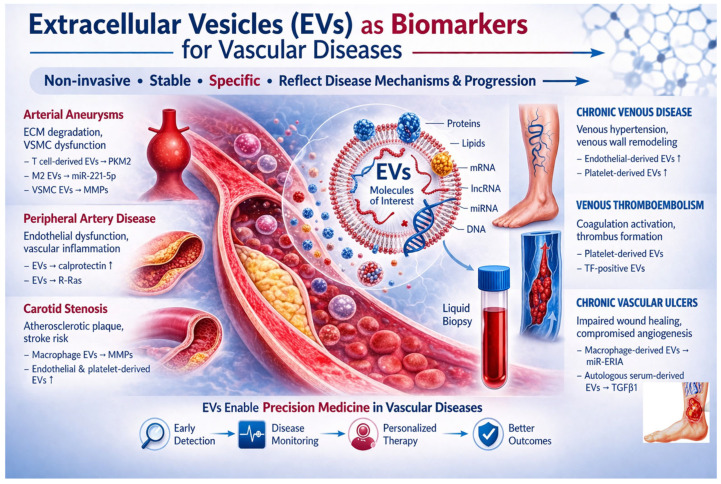
Role of Extracellular Vesicles in Vascular Diseases.

Despite these limitations, the rapid expansion of EV research suggests that these vesicles may play an increasingly important role in the future of vascular medicine. Continued interdisciplinary collaboration between clinicians, molecular biologists, and bioengineers will be essential for translating EV-based discoveries into practical diagnostic and therapeutic applications [107] (Figure 1).

## 10. Conclusions

Extracellular Vesicles have emerged as key mediators of intercellular communication and important contributors to the pathophysiology of vascular diseases. Growing evidence indicates that EVs are involved in multiple biological processes relevant to vascular pathology, including inflammation, endothelial dysfunction, thrombosis, extracellular matrix remodeling, and angiogenesis.

Alterations in EV concentration, cellular origin, and molecular cargo have been described in a wide range of vascular disorders, including arterial aneurysms, peripheral artery disease, carotid stenosis, chronic venous disease, venous thromboembolism, and chronic vascular ulcers. These findings suggest that EVs may serve as valuable biomarkers for disease detection, risk stratification, and monitoring of disease progression.

In addition to their diagnostic potential, EVs are increasingly being investigated as therapeutic tools due to their capacity to deliver bioactive molecules and modulate cellular responses in target tissues. Although several methodological and technical challenges remain, ongoing advances in EV biology and analytical technologies are expected to accelerate the translation of EV-based strategies into clinical practice.

Overall, EVs represent a promising frontier in vascular research and may contribute to the development of more precise diagnostic tools and innovative therapeutic approaches for vascular diseases.

However, it should be acknowledged that the majority of current evidence is derived from experimental and exploratory studies, and the translation of EV-based applications into clinical practice remains in its early stages. Future research should focus on large-scale validation studies, methodological standardization, and the integration of EV-based biomarkers into existing clinical frameworks to fully realize their potential in vascular medicine.

## Figures and Tables

**Table 1:** Methods used for the isolation and characterization of extracellular vesicles.

Method	Principle	Advantages	Limitations	Typical Applications
Differential Ultracentrifugation	Sequential centrifugation at increasing speeds to separate EVs by size and density	Widely used; considered reference method; suitable for large volumes	Time-consuming; requires specialized equipment; co-isolation of protein aggregates and lipoproteins	General EV isolation from plasma, serum, cell culture
Density Gradient Ultracentrifugation	Separation based on buoyant density using sucrose or iodixanol gradients	Higher purity than standard ultracentrifugation; better separation of EV subtypes	Labor-intensive; low yield; technically demanding	Isolation of highly purified EV populations
Size-Exclusion Chromatography (SEC)	Separation based on particle size through porous columns	Preserves EV integrity; high purity; reproducible	Lower yield; dilution of samples; requires concentration steps	Clinical samples; proteomic and RNA analysis
Polymer-based Precipitation	Use of polymers (e.g., PEG) to precipitate EVs from solution	Simple; fast; scalable; no need for specialized equipment	Low specificity; co-precipitation of contaminants; not ideal for downstream functional studies	High-throughput screening; preliminary analyses
Immunoaffinity Capture	Use of antibodies targeting EV surface markers (e.g., CD63, CD81)	High specificity; allows isolation of EV subpopulations	Expensive; limited yield; depends on marker availability	Biomarker discovery; cell-specific EV studies
Nanoparticle Tracking Analysis (NTA)	Tracks Brownian motion to determine particle size and concentration	Quantitative; widely used; provides size distribution	Cannot distinguish EV subtypes; sensitive to contaminants	EV quantification and size analysis
Dynamic Light Scattering (DLS)	Measures light scattering fluctuations to estimate particle size	Rapid and simple	Less accurate for heterogeneous samples; biased toward larger particles	Preliminary size characterization
Flow Cytometry	Detection of EVs labeled with fluorescent antibodies	Allows phenotyping and cell-origin identification	Limited sensitivity for small EVs; requires optimization	Surface marker analysis; clinical studies
Transmission Electron Microscopy (TEM)	Direct visualization of EV morphology at high resolution	Gold standard for structural analysis	Time-consuming; qualitative; low throughput	Morphological validation of EVs
Western Blotting	Detection of EV-associated proteins (e.g., CD9, CD63, TSG101)	Confirms EV identity; widely accepted	Semi-quantitative; requires prior isolation	Validation of EV markers
Omics Approaches (Proteomics, RNA-seq)	High-throughput analysis of EV molecular cargo	Comprehensive molecular profiling; biomarker discovery	Expensive; requires specialized expertise; data complexity	Identification of EV-based biomarkers

**Table 2:** Extracellular vesicle (EV) cargo and their potential roles in vascular diseases.

EV Cargo Type	Representative Molecules	Cellular Source of EVs	Associated Vascular Diseases	Biological Role	Remarks
MicroRNAs (miRNAs)	miR-221-5p; miR-ERIA; disease-specific miRNA signatures	Macrophages (M2), endothelial cells, VSMCs, stem cells	Abdominal aortic aneurysm (AAA), peripheral artery disease (PAD), carotid stenosis, chronic vascular ulcers	Regulation of inflammation, macrophage polarization, angiogenesis, and VSMC phenotypic switching	High stability in circulation; promising biomarkers but limited disease specificity due to overlap across conditions
Proteins (pro-inflammatory)	Calprotectin	Leukocytes, inflammatory cells	PAD	Promotion of vascular inflammation and immune activation	Correlates with disease severity and prognosis; potential biomarker candidate
Proteins (matrix remodeling)	Matrix metalloproteinases (MMPs)	VSMCs, macrophages	AAA, carotid atherosclerosis	Extracellular matrix degradation and vascular wall weakening	Mechanistically relevant; associated with plaque instability and aneurysm progression
Proteins (metabolic enzymes)	Pyruvate kinase muscle isozyme 2 (PKM2)	T lymphocytes	AAA	Modulation of macrophage metabolism, oxidative stress, and lipid peroxidation	Highlights EV-mediated immune-metabolic interactions in vascular pathology
Proteins (vascular regulators)	R-Ras	Endothelial cells	PAD	Regulation of endothelial function, vascular stability, and angiogenesis	Levels correlate with disease severity; potential minimally invasive biomarker
Procoagulant factors	Tissue factor (TF); procoagulant phospholipids (PPL)	Platelets, endothelial cells, tumor cells	Venous thromboembolism (VTE)	Activation of coagulation cascade and thrombin generation	Strong mechanistic role; variable predictive value across clinical studies
Cytokines/inflammatory mediators	Various cytokines	Immune cells, endothelial cells	Chronic venous disease (CVD), PAD, carotid stenosis	Amplification of inflammatory signaling and endothelial dysfunction	Limited specificity; better suited for multi-marker panels
Regenerative cargo (stem cell-derived EVs)	Pro-angiogenic miRNAs; growth factors	Adipose-derived stem cells (ADSCs), mesenchymal stem cells (MSCs), endothelial progenitor cells (EPCs)	PAD, chronic vascular ulcers	Promotion of angiogenesis, tissue repair, and re-epithelialization	Primarily therapeutic application; promising for regenerative medicine

## Data Availability

All data generated or analyzed during this study are included in this published article.

## Abbreviations

The following abbreviations are used in this manuscript:

AAA	Abdominal aortic aneurysm
ADSCs	Adipose-Derived Stem Cells
AGEs	Advanced Glycation End Products
CVD	Chronic Venous Disease
CVUs	Chronic Venous Ulcers
DFUs	Diabetic Foot Ulcers
DLS	Dynamic light scattering
EPCs	Endothelial progenitor cells
EVs	Extracellular vesicles
ISEV	International Society for Extracellular Vesicles
miRNAs	MicroRNAs
MMPs	Matrix metalloproteinases
mRNA	Messenger RNA
MVBs	Multivesicular bodies
NTA	Nanoparticle tracking analysis
PAD	Peripheral Artery Disease
PKM2	Pyruvate Kinase Muscle Isozyme 2
PPL	Procoagulant Phospholipids
ROC	Receiver operating characteristic
SEC	Size-Exclusion Chromatography
s-EVs	Serum-derived Extracellular Vesicles
TAA	Thoracic Aortic Aneurysm
TEM	Transmission Electron Microscopy
TF	Tissue Factor
TGFβ1	Transforming Growth Factor-beta 1
VD	Vascular disease
VSMCs	Vascular smooth muscle cells
VTE	Venous Thromboembolism
